# SENP1-Mediated Desumoylation of DBC1 Inhibits Apoptosis Induced by High Glucose in Bovine Retinal Pericytes

**DOI:** 10.1155/2016/6392658

**Published:** 2016-03-27

**Authors:** Jian Gao, Xia Chen, Qing Gu, Xiaoxiao Liu, Xun Xu

**Affiliations:** Department of Ophthalmology, Shanghai First People's Hospital, Shanghai Jiao Tong University, School of Medicine, Shanghai 200080, China

## Abstract

Pericyte loss is an early characteristic change in diabetic retinopathy, but its precise molecular mechanisms have not been elucidated. This study investigated the role of SENP1 in pericyte loss in diabetic retinopathy. We demonstrated that a high concentration of glucose inhibited the expression of the Sentrin/SUMO-specific protease 1 (SENP1), which resulted in an increase in DBC1 sumoylation in bovine retinal pericytes (BRPCs). Furthermore, SENP1 overexpression attenuated hyperemia-induced apoptosis of BPRCs, and SENP1 knockdown aggravated this effect. We also provide evidence that DBC1 sumoylation/desumoylation is involved in the SENP1-regulated apoptosis of BRPCs under high glucose conditions. Understanding the role of SENP1 in the pathogenesis of high glucose induced pericyte loss could help elucidate important targets for future pharmacological interventions.

## 1. Introduction

Diabetic retinopathy (DR) is the leading cause of visual loss in working-aged people and the most common microvascular complication in diabetic patients, despite the recent improvement in the management of DR via glycemic control and photocoagulation [1, 2]. Pericyte loss is one of the earliest and most characteristic changes in DR [3, 4]. Thus, preventing pericyte loss for the primary prevention of DR would be beneficial. However, the mechanisms by which hyperglycemia leads to pericyte loss remain largely unknown.

In recent years, sumoylation has emerged as an important mechanism in the regulation of multiple cellular signaling pathways [5, 6]. Sumoylation is catalyzed by the activation (E1), conjugation (E2), and ligation (E3) of enzymes [5]. It can be reversed by a family of Sentrin/SUMO-specific proteases (SENPs) [7]. Among these family members, SENP1 is able to deconjugate either SUMO1 or SUMO2/3 modified proteins [8, 9]. SENP1 has been reported to play an important role in the control of DNA damage responses and cellular apoptosis [10, 11]. However, the role of SENP1 in diabetic vascular complications has not been investigated.

DBC1 (deleted in breast cancer) was initially identified as a putative tumor suppressor [12]. DBC1 modification by Small Ubiquitin-like Modifier 2/3 (SUMO2/3) is crucial for cellular apoptosis under genotoxic stress [13]. However, it remains unknown whether high glucose is involved in the regulation of DBC1 sumoylation.

In the present study, we demonstrated that high glucose induces the sumoylation of DBC1 via the inhibition of SENP1 expression in bovine retinal pericytes (BRPCs). Knockdown of SENP1 promoted hyperglycemia-induced BRPC apoptosis, whereas the overexpression of SENP1 inhibited it. These results indicate that modification of DBC1 by SENP1 contributes to BRPC apoptosis in response to high glucose.

## 2. Materials and Methods

All chemicals were reagent-grade quality and were purchased from Sigma Chemicals (St. Louis, MO, USA), unless stated otherwise.

### 2.1. Cells

BRPCs were isolated from fresh bovine eyes in accordance with a previous study [14]. The cells were cultured in Dulbecco's Modified Eagle's Medium (DMEM, Gibco, USA) supplemented with 10% fetal bovine serum (FBS, Gibco, USA), at 37°C, with 5% CO_2_. The medium was changed every two days. The cells were identified using the anti-*α* isoform of smooth-muscle actin (Sigma-Aldrich Corporation, St. Louis, MO, USA). Cells cultured for more than three passages were used in further high glucose treatment and transfection. For high glucose treatment, the cells were exposed to either high glucose (30 mmol/L; HG) or normal glucose (5 mmol/L; NG) for 12–48 h.

### 2.2. Cell Transfection

The HA-SENP1 plasmid (HA-SENP1) was created via PCR cloning into the HindIII and Xbal I sites of the pcDNA3.0-HA vector. The primers of SENP1 were 5′-ATGGATGATATTGCTGATAGGATG-3′ and 5′-TCACAAGAGTTTTCGGTGGAGG-3′. The pcDNA3.0 vector (pcDNA) was used as a control empty plasmid. The Flag-DBC1 plasmid (Flag-DBC1) was created by PCR cloning into the HindIII and Kpn I sites of the pcDNA3.0-Flag vector. The primers for DBC1 were 5′-ATGTCCCAGTTTAAGCGCCAGC-3′ and 5′-TCAGTTGCTAGGTGCCGGCTC-3′. The ablation of SENP1 was performed via the transfection of the BRPC with SiRNA-SENP1 (Si-SENP1) as follows: 5′-GGCTCAATGACGAGATCATTT-3′. The ablation of DBC1 was performed by transfection of the BRPC with SiRNA-DBC1 (Si-DBC1), 5′-AAACGGAGC-CUACUGAACAUU-3′. The nonspecific siRNA was used as a control (Si-NC). The pcDNA3.0-Flag-DBC1 mutant (K591R) construct was designed to mutate (Flag-DBC1 K591R); the construct was prepared through site-directed mutagenesis based on the wild-type construct by using a QuikChange Mutagenesis Kit (Agilent, USA). The primers used for the one-site mutation (K591R) were 5′-CCAAGGAGGAAGAAGCCATCAGAGAGGAGGTGG-3′ and 5′-CCACCTCCTCTCTGATGGCTTCCTCCTTGG-3′. Cells were transfected by Lipofectamine 2000 (Invitrogen, USA) following the protocol provided by the manufacturer.

### 2.3. Animals

Eight-week-old male Sprague-Dawley rats weighing ~200 g each (Shanghai Laboratory Animal Center, Chinese Academy of Sciences) were randomly selected for intraperitoneal administration of either 65 mg/kg streptozotocin (STZ) or citrate buffer alone. Rats were categorized as diabetic when their blood glucose exceeded 16.7 mmol/L at 48 h after STZ administration. Blood glucose levels were 4.6–6.0 mmol/L in nondiabetic rats and 18.7–29.8 mmol/L in diabetic rats. After 8 weeks, the eyes were resected from deeply anesthetized animals and prepared for Western blot analysis.

### 2.4. Western Blotting

Fifty micrograms of protein obtained from each sample (BRPCs) was subjected to sodium dodecyl sulfate-polyacrylamide gel electrophoresis (SDS-PAGE) in a Bio-Rad miniature slab gel apparatus and electrophoretically transferred onto a nitrocellulose membrane. The membrane was blocked in a 5% nonfat dried-milk solution and incubated overnight with rabbit anti-SENP1 (ab108981, 1 : 1000, Abcam, USA), rabbit anti-PARP (#9542, 1 : 1000, Cell Signaling Technology, USA), rabbit anti-BCL-2 (#2870, 1 : 1000, Cell Signaling Technology, USA), mouse anti-HA (ab18181, 1 : 1000, Abcam, USA), or Flag antibody (F1804, 1 : 1000, Sigma, USA). Blots were stained with mouse anti-*β*-actin (A5441, 1 : 1000, Sigma, USA) (as an internal control) to confirm equivalent total-protein loading.

### 2.5. Real-Time RT-PCR

Total RNA was extracted from BRECs and rat retinal tissue using TRIZOL reagent (Invitrogen Life Technologies, Gaithersburg, MD) and stored at −80°C. The DyNAmo Flash SYBR Green qPCR kit (Finnzymes Oy, Espoo, Finland) was used according to the manufacturer's instructions. The primer sequences (sense/antisense) used were as follows: SENP1 (BRECs), 5′-CATGCCATTTTCCACCGTGTT-3′/5′-AAGGCATGTGGAGGAAGAGTG-3′; SENP1 (Rat), 5′-AGTGAAACGCTGGACAAA-3′/5′-CCTGGGCTTTGGTTTGGA-3′; *β*-actin, 5′-GCACCGCAAATGCTTCTA-3′/5′-GGTCTTTACGGATGTCAACG-3′. The specificity of the amplification product was determined by a melting curve analysis. Standard curves were generated for the expression of each gene by preparing serial dilutions with known quantities of each cDNA template. Relative quantification of the signals was performed by normalizing the signals of different genes with the *β*-actin signal. Signal intensities in the control lanes were arbitrarily assigned a value of 1.0.

### 2.6. Immunoprecipitation Assay

Cell lysates were incubated with an appropriate antibody overnight at 4°C, followed by incubation with Protein A/Protein G-coated agarose beads (Millipore, Massachusetts, USA) for another 4 h at 4°C. After samples were washed four times with ice-cold IP buffer and supernatants were removed by centrifugation at 2,000 ×g for 1 min, proteins were coprecipitated. The proteins were then separated from the beads using an immunoprecipitation loading buffer for 15 min at 95°C. The supernatants were collected and subjected to SDS-PAGE followed by immunoblot analysis using antibodies against RH, SUMO2/3, DBC1, HA, Flag, and SENP1.

### 2.7. Flow Cytometry for the Measurement of Apoptosis

Apoptosis was assessed by using the Annexin V/propidium iodide (PI) kit, according to the manufacturer's instructions (Bender Med Systems, USA). BRPCs (5 × 10^5^) were washed twice with PBS and suspended in 90 *μ*L 1x binding buffer. A total of 4.0 *μ*L of Annexin V FITC were added to the cell suspension, vortexed, and incubated for 10 min in the dark. Then 4.0 *μ*L PI was added to the cell suspension and vortexed. Finally, 60 *μ*L of 1x binding buffer was added, and samples were evaluated using flow cytometry.

### 2.8. Statistical Analysis

The intensities of relative proteins were measured using ImageJ software (National Institutes of Health) [15]. Statistical analyses were performed using the standard two-tailed Student's *t*-test, assuming unequal variances. Prism 4.0 software system (GraphPad, San Diego, CA, USA) and the statistical software program SPSS version 17.0 for Windows (SPSS, Chicago, IL, USA) were used for these analyses. *P* values < 0.05 were considered significant in all cases.

## 3. Results

### 3.1. The BRPCs in Culture and Identification

**Figure 1:**
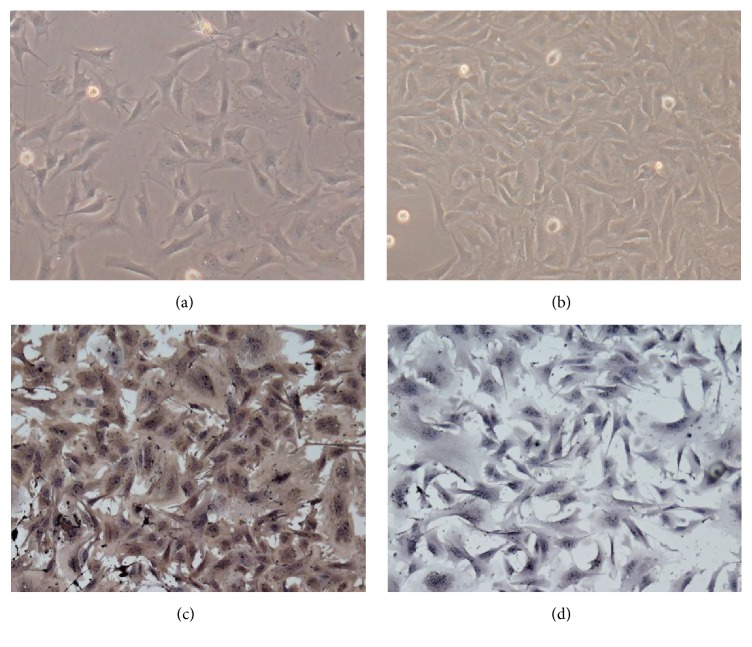
(a) Phase-contrast microscopy of BRPCs at five days after isolation, with irregular peripheries ×100. (b) Phase-contrast microscopy of BRPCs at 10 days after isolation, with non-contact-inhibited and overlapping pattern of growth ×100. (c) Positive staining of pericytes with anti-*α* isoforms of smooth-muscle actin (DAB ×200). (d) Negative staining of pericytes with anti-von Willebrand factor (DAB ×200).

Pericytes may be identified by morphological criteria and also through analysis of antigen expression detected by immunofluorescence with specific antibodies. As the previous study demonstrated, the BRPCs showed to be characterized by highly irregular peripheries and phase-dense filaments at five days after isolation (Figure 1(a)) [14, 16]. At 10 days after isolation, the BRPCs showed a non-contact-inhibited and overlapping pattern of growth at confluence (Figure 1(b)). Immunohistochemical staining showed positive staining for anti-*α* isoforms of smooth-muscle actin (Figure 1(c)) and negative staining for von Willebrand factor (Figure 1(d)) [14, 16].

### 3.2. High Glucose Inhibited SENP1 Expression in BRPCs and Retinas

**Figure 2:**
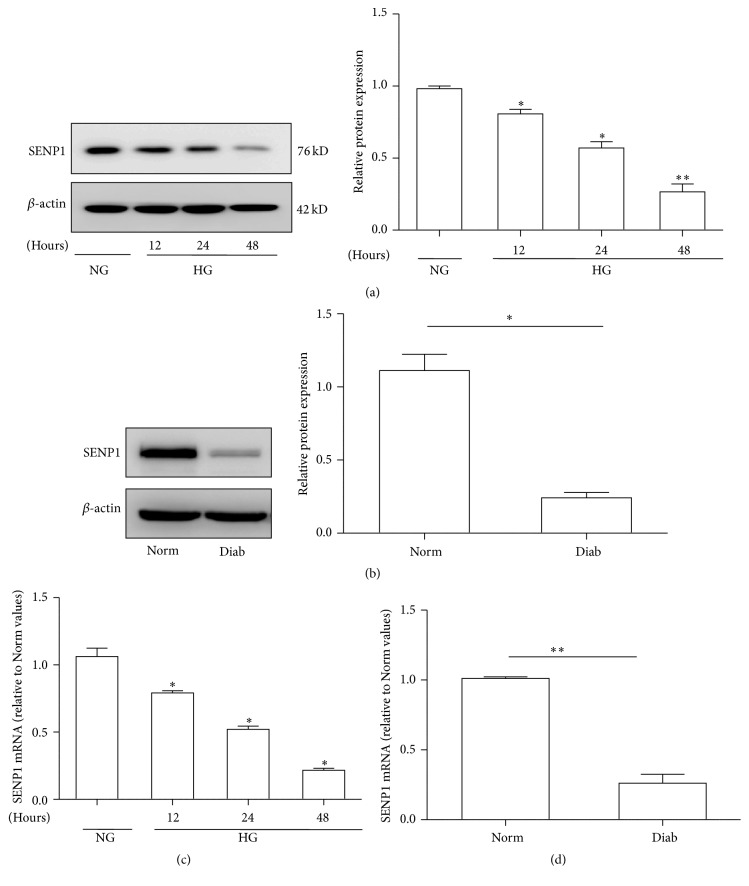
High glucose inhibited SENP1 expression in BRPCs and retinas. (a) Western blot analyses of SENP1 protein expression in BRPCs cultured in normal glucose (NG) and high glucose (HG) (12 h, 24 h, and 48 h). (b) Western blot analyses of SENP1 protein expression in the retinas of normal (Norm) and diabetic (Diab) rats. (c) Analyses of SENP1 mRNA in BRPCs cultured in normal glucose (NG) and high glucose (HG) (12 h, 24 h, and 48 h). (d) Analyses of SENP1 mRNA in the retinas of normal (Norm) and diabetic (Diab) rats. Data are presented as their means ± SEM. Bars indicate SDs. ^*∗*^ *P* < 0.05 versus NG or Norm; ^*∗∗*^ *P* < 0.01 versus NG or Norm.

As shown in Figure 2(a), treatment with high glucose resulted in a decrease of SENP1 expression; the levels of mRNA and protein were lower in the HG groups than in the NG group in BRPCs. Of note, we found that the effects of high glucose on SENP1 expression were time dependent. We also examined the levels of SENP1 mRNA and protein in the retinas of control rats (Norm) and rats that maintained hyperglycemia for eight weeks (Diab). We found that hyperglycemia caused a significant decrease in the mRNA and protein of SENP1 (Figure 2(b)).

### 3.3. SENP1 Played an Antagonistic Role in BRPC Apoptosis under High Glucose Conditions

**Figure 3:**
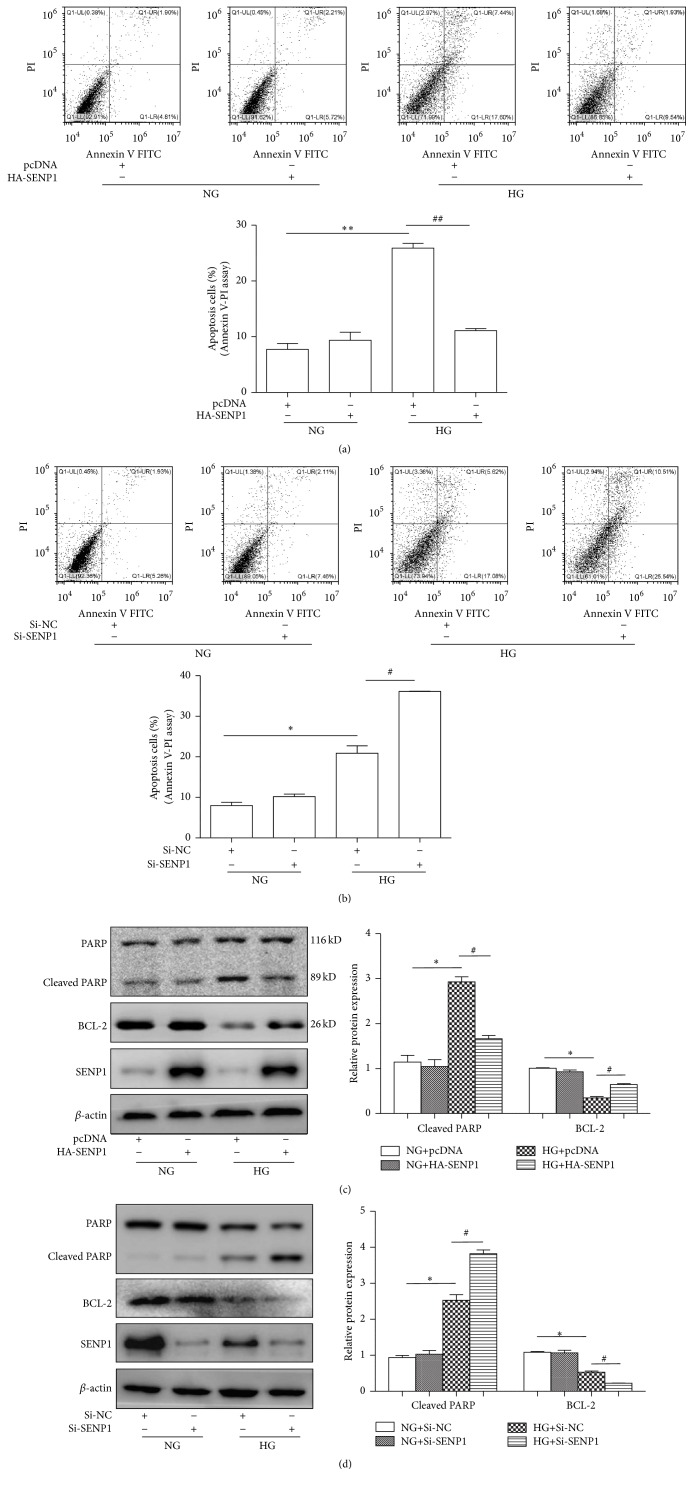
SENP1 played an antagonistic role in BRPC apoptosis under high glucose conditions. (a) Analysis of cellular apoptosis levels in the BRPCs treatment groups for 48 h: NG+pcDNA, NG+HA-SENP1, HG+pcDNA, and HG+HA-SENP1. (b) Analysis of cellular apoptosis levels in the BRPCs treatment groups for 48 h: NG+Si-NC, NG+Si-SENP1, HG+Si-NC, and HG+Si-SENP1. (c) SENP1, Cleaved PARP, and BCL-2 protein expression profiles in the four groups for 48 h: NG+pcDNA, NG+HA-SENP1, HG+pcDNA, and HG+HA-SENP1. (d) SENP1, Cleaved PARP, and BCL-2 protein expression profiles in the four groups for 48 h: NG+Si-NC, NG+Si-SENP1, HG+Si-NC, and HG+Si-SENP1. Data are presented as their means ± SEM. Bars indicate SDs. ^*∗*^ *P* < 0.05 versus NG+Si-NC or NG+pcDNA; ^#^ *P* < 0.05 versus HG+Si-NC or HG+pcDNA; ^*∗∗*^ *P* < 0.01 versus NG+pcDNA; ^##^ *P* < 0.01 versus HG+pcDNA.

In an attempt to determine the effects of SENP1 on the apoptosis of BRPCs under high glucose conditions, we overexpressed SENP1 in BRPCs. In these experiments, cells were transfected with pcDNA (control vector) or the HA-SENP1 and exposed to normal or high glucose for 48 h. Pericyte apoptosis was assessed via Annexin V/PI flow cytometric analysis. The apoptosis of BRPC incubated under high glucose conditions was significantly greater than under normal glucose conditions, and this hyperglycemia-induced increase in cell apoptosis was attenuated by SENP1 overexpression (Figure 3(a)). We also knocked down SENP1 in BRPCs. In these experiments, cells were transfected with Si-NC or the Si-SENP1 and exposed to normal or high glucose for 48 h. Knockdown of SENP1 led to an increase in the apoptotic cells in the presence of high glucose, but not in its absence (Figure 3(b)). It was consistent with the results in previous study [13]. We also investigated whether SENP1 affected apoptosis-associated gene expression, such as cleaved poly-ADP ribose polymerase (Cleaved PARP) [17] and BCL-2 [18]. The levels of the two proteins in the SENP1 overexpression (Figure 3(c)) or knockdown (Figure 3(d)) cells exposed to normal or high glucose, respectively, were determined by Western blotting. The expression trends of the two proteins were found to be consistent with the flow cytometric results. These data suggest that SENP1 plays an antagonistic role in pericyte apoptosis under high glucose conditions.

### 3.4. High Glucose Induced DBC1 Sumoylation in BRPCs

**Figure 4:**
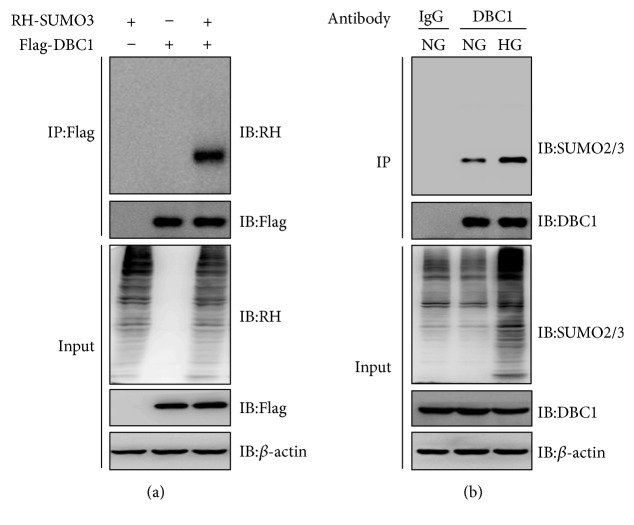
High glucose induced DBC1 sumoylation in BRPCs. (a) After HEK293T cells were transfected with Flag-DBC1, with or without RH-SUMO3 as indicated for 48 h, cell lysates were subjected to immunoprecipitation with anti-Flag antibody followed by immunoblot analysis. (b) BRPCs were treated with normal (NG) or high glucose (HG) for 48 h. Their lysates were subjected to immunoprecipitation with IgG or anti-DBC1 antibody followed by immunoblot analysis.

Further, we identified the mechanism that mediates SENP1-regulated pericyte apoptosis under high glucose. Because DBC1 sumoylation has been implicated in SENP1-mediated apoptosis, we examined whether high glucose induced DBC1 sumoylation. Therefore, we first examined whether DBC1 can be sumoylated upon overexpression of SUMO3. As expected, we found that DBC1 was modified by SUMO3 (Figure 4(a)). Since sumoylation has been implicated in DNA damage response [19, 20], we examined whether hyperglycemia stress induces DBC1 sumoylation. We found that treatment of BRPC with high glucose led to an increase in SUMO3 modification of endogenous DBC1 (Figure 4(b)).

### 3.5. SENP1 Inhibited DBC1 Sumoylation Induced by High Glucose

**Figure 5:**
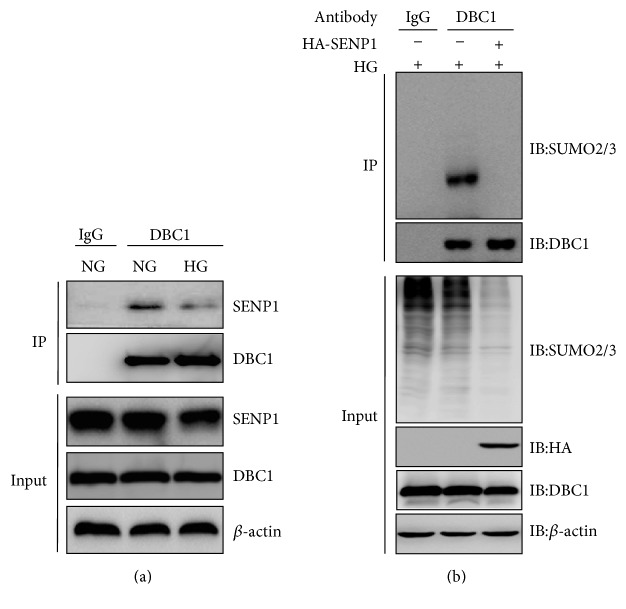
SENP1 inhibited the DBC1 sumoylation induced by high glucose. (a) BRPCs incubated with normal (NG) or high glucose (HG) for 48 h were subjected to immunoprecipitation with IgG or anti-DBC1 antibody followed by immunoblot analysis. (b) SENP1 was overexpressed in BRPCs under high glucose for 48 h. Their lysates were subjected to immunoprecipitation with IgG or anti-DBC1 antibody followed by immunoblot analysis.

To determine whether SENP1 was the desumoylating enzyme of DBC1, we immunopurified DBC1-associated proteins (anti-DBC1). Western blotting with an anti-SENP1 antibody revealed the association of SENP1 with DBC1, and this interaction was markedly reduced by high glucose treatment (Figure 5(a)). To determine whether SENP1 is indeed capable of desumoylating DBC1, SENP1 was overexpressed in BRPCs under high glucose. SENP1 overexpression caused a marked reduction in DBC1 sumoylation which was induced by high glucose (Figure 5(b)).

### 3.6. Sumoylation of DBC1 Contributed to SENP1-Mediated Apoptosis under High Glucose Conditions

**Figure 6:**
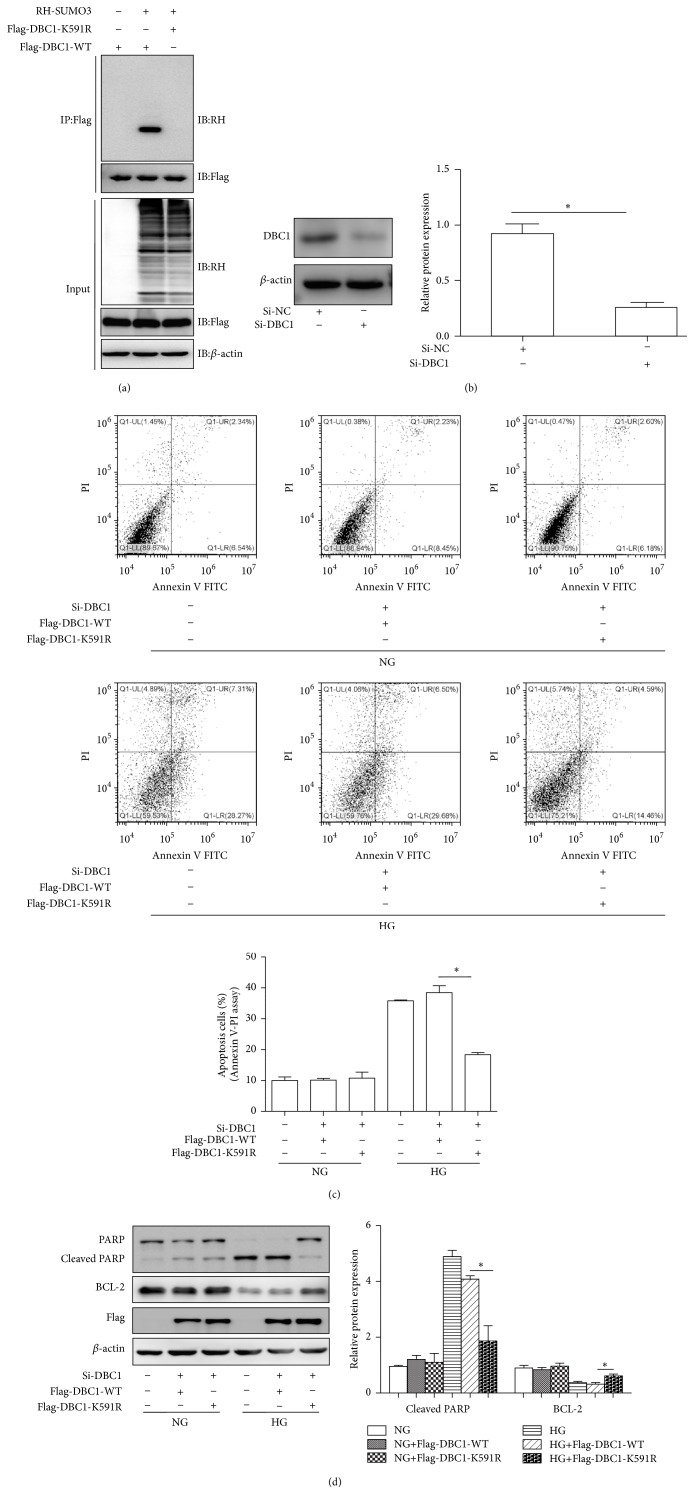
Sumoylation of DBC1 contributed to SENP1-mediated apoptosis under high glucose conditions. (a) After HEK293T cells were transfected with Flag-DBC1-WT or Flag-DBC1-K591R, with or without RH-SUMO3 as indicated for 48 h, cell lysates were subjected to immunoprecipitation with anti-Flag antibody followed by immunoblot analysis. (b) Effect of siRNA-mediated knockdown on DBC1 expression in BRPCs incubated in normal glucose. (c) Analysis of cellular apoptosis levels in the BRPC treatment groups for 48 h: NG, NG+Flag-DBC1-WT, NG+Flag-DBC1-K591R, HG, HG+Flag-DBC1-WT, and HG+Flag-DBC1-K591R. (d) Cleaved PARP and BCL-2 protein expression profiles in the six groups. Data are presented as their means ± SEM. Bars indicate SDs. ^*∗*^ *P* < 0.05 versus HG+Flag-DBC1 WT.

To determine whether DBC1 sumoylation was responsible for SENP1-mediated apoptosis under high glucose conditions, two plasmids that expressed either the wild-type (Flag-DBC1-WT) or the DBC1 mutant (Flag-DBC1-K591R) were constructed. Since Lys591 had been reported as the main SUMO acceptor site, the Lys residues were substituted by Arg. The K591R mutation completely prevented DBC1 sumoylation (Figure 6(a)). These plasmids were used for transfecting BRPCs in which endogenous DBC1 expression was knocked down using siRNAs and the cells were exposed to normal or high glucose for 48 h. The knockdown effect of the DBC1 siRNA in BRPCs was assessed using Western blot analysis (Figure 6(b)). Consistently, Flag-DBC1-WT, but not the Flag-DBC1-K591R, increased the apoptosis of BRPC under high glucose (Figure 6(c)). The expression trend of the Cleaved PARP and BCL-2 was found to be consistent with the flow cytometric results (Figure 6(d)). This was similar to what was observed in BRPCs transfected with HA-SENP1. These results indicated that DBC1 sumoylation is a crucial step in SENP1-mediated apoptosis under high glucose conditions.

## 4. Discussion

Sumoylation is a highly transient posttranslational protein modification that affects the cell cycle, proliferation, apoptosis, and differentiation [21]. SENPs, which remove SUMO intact from their substrates, are the central players in the regulation of protein/sumoylation balances [22]. In the current study, we provided evidence that DBC1 sumoylation increased as a result of the hyperglycemia-induced inhibition of SENP1 expression in BRPCs. Overexpression of SENP1 conferred resistance to the apoptosis of BRPCs induced by high glucose via desumoylating DBC1.

To our knowledge, our study is the first to report that high glucose inhibits SENP1 expression in BRPCs. Sumoylation/desumoylation is a highly dynamic process that regulates various physiological processes, including cell growth, differentiation, senescence, oxidative stress, and apoptosis [23]. Previously, the SENP1-mediated protein sumoylation has been demonstrated to play a key role in pancreatic immune responses, *β*-cell damage, and, consequently, diabetes progression [24]. SENP1 has been shown to be a molecular mechanism for the pathogenesis of diabetes, and it may help to develop a novel therapeutic strategy for the treatment of diabetes. However, the role of SENP1 in the pathogenesis of diabetic vascular complications is an important question that has never been answered. We sought to investigate the roles and detailed mechanisms by which SENP1 regulates the apoptosis of BRPC under high glucose conditions. We developed SENP1 siRNAs and detected their silence efficiency in BRPCs. Importantly, SENP1 siRNA significantly aggravated the apoptosis of BRPCs induced by hyperglycemia stress, whereas the overexpression of SENP1 inhibited cellular apoptosis.

The mechanisms by which SENP1 suppresses apoptosis reactions under high glucose conditions are poorly understood. The discovery of DBC1 as a modulator of SIRT1, HDAC3, and several nuclear receptors raises the possibility that DBC1 may be a master regulator of the interconnection between metabolism and epigenetics [25, 26]. The study of this multifunctional protein is likely to provide novel understanding and potential new therapeutic options for multiple human diseases and conditions such as obesity, metabolic syndrome, atherosclerosis, inflammation, aging, and cancer [27]. DBC1 sumoylation could cause a dramatic increase in DBC1-SIRT1 interactions, which could lead to the release of p53 from SIRT1 for transcriptional activation [13]. This study demonstrated that the treatment of BRPCs with high glucose led to an increase in SUMO-modification of endogenous DBC1. We also found that SENP1 overexpression could inhibit the DBC1 sumoylation induced by high glucose, and DBC1 WT (though not the K591R mutant) increased the apoptosis of BRPCs. Together, these results suggest that there may be causal relationships between the reduced levels of cellular apoptosis mediated by SENP1 and the sumoylation levels of DBC1.

## 5. Conclusion

In conclusion, our results provide molecular insight into the regulation of the apoptosis induced by high glucose in BRPCs. We provide evidence that high glucose inhibits SENP1 expression. We also demonstrated the crucial role of SENP1 in the control of apoptosis, through a DBC1 sumoylation-dependent pathway. Our data suggest that SENP1 could be a potential therapeutic target to prevent pericytes loss in DR.
